# Laparoscopic repair of hepatic herniation through a ventral incisional hernia: a case report

**DOI:** 10.1186/s13256-021-02682-z

**Published:** 2021-02-12

**Authors:** Toshihiro Misumi, Masahiro Nishihara, Keizo Sugino, Yukari Kawasaki

**Affiliations:** 1 Department of Surgery, Tsuchiya General Hospital, 3-30 Nakajimacho, Naka-ku, Hiroshima, 730-8655 Japan; 2 Department of Gastroenterological Surgery, Hiroshima Prefectural Hospital, 1-5-54 Ujina-Kanda, Minami-ku, Hiroshima, 734-8530 Japan

**Keywords:** Body mass index, Cardiac surgical procedures, Ventral hernia, Median sternotomy, Hepatic herniation, Laparoscopic repair, Intraperitoneal underlay mesh, Case report

## Abstract

**Background:**

Ventral incisional hernia is a common problem after abdominal surgery. Most patients with these hernias present with greater omentum and gastrointestinal prolapse. However, hepatic herniation through a ventral incisional hernia is a rare phenomenon that has been seldom reported in the literature. We report the case of a ventral incisional hernia with hepatic herniation treated with laparoscopic repair.

**Case presentation:**

A 68-year-old Japanese women with a history of myocardial resection for hypertrophic cardiomyopathy 1 year earlier was admitted to our hospital with symptoms of vomiting and epigastric pain. Physical examination showed a 4-cm epigastric mass. Abdominal computed tomography revealed left hepatic lobe herniation through the lower edge of a mid-sternal incision. We diagnosed the patient with a ventral incisional hernia with hepatic herniation. The patient underwent laparoscopic hernia repair. During an 18-month follow-up, no recurrence or symptoms have been observed.

**Conclusions:**

To the best of our knowledge, this is the first case report of laparoscopic repair of ventral incisional hernias with hepatic herniation. Laparoscopic repair was useful and suitable for this rare herniation due to its minimally invasive nature and ability to achieve sufficient visibility of the surgical field. Laparoscopic repair could be a potential treatment option for elective surgery for this disease, which is often treated conservatively.

## Background

Ventral incisional hernias are a common problem after abdominal surgery [1]. Most patients with this hernia present with greater omentum and gastrointestinal prolapse. However, herniation of abdominal parenchymal organs such as the liver is a very uncommon situation [2]. Cases of liver prolapse typically occur as congenital or traumatic diaphragmatic hernias; their association with ventral incisional hernia is extremely rare. These herniations cause nausea, discomfort, and sometimes severe liver dysfunction, which may require surgical repair in some cases. We report the case of a ventral incisional hernia with hepatic herniation that was treated with laparoscopic repair.

## Case presentation

**Fig. 1:**
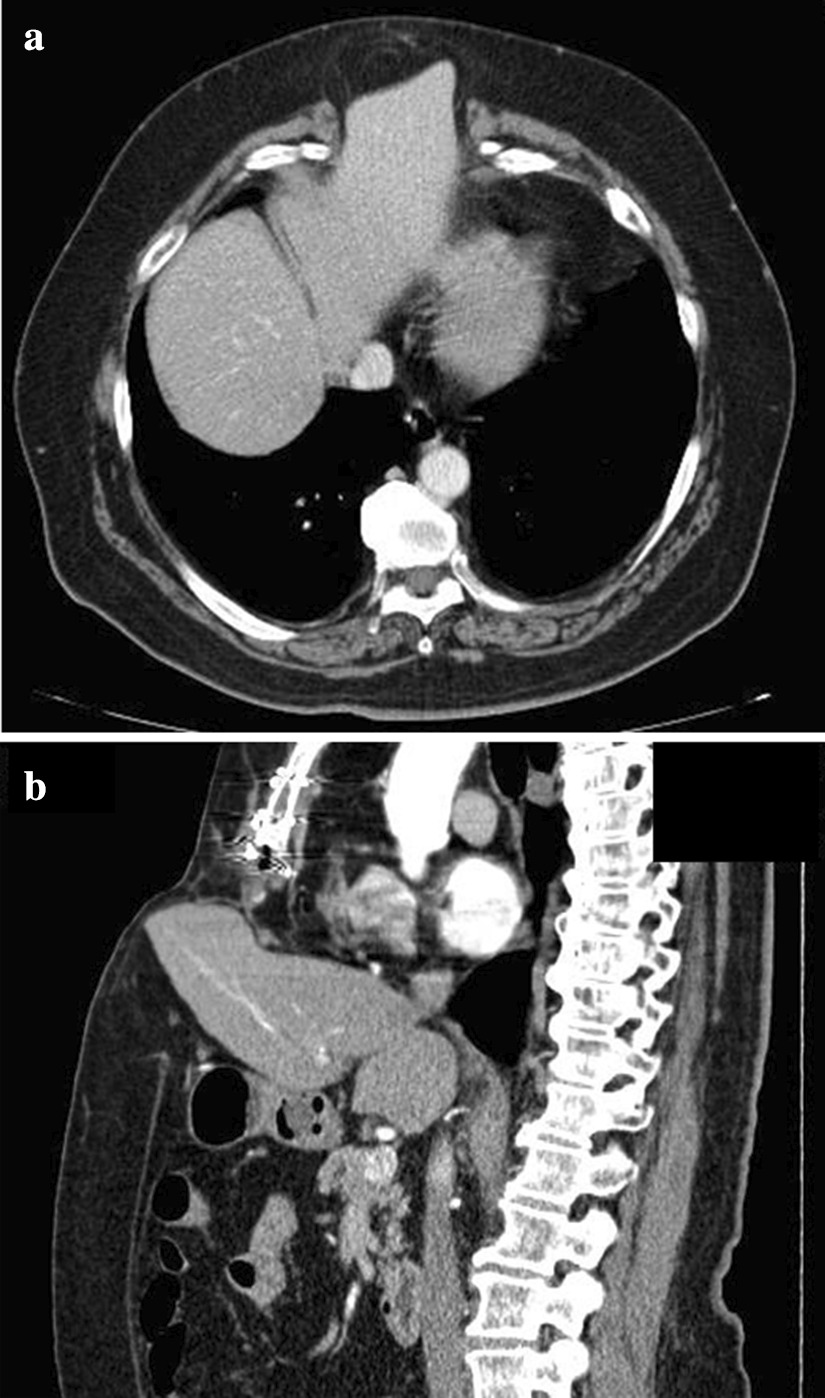
Abdominal computed tomography showing left hepatic lobe herniation through an incisional hernia. **a** Axial view. **b** Sagittal view

**Fig. 2:**
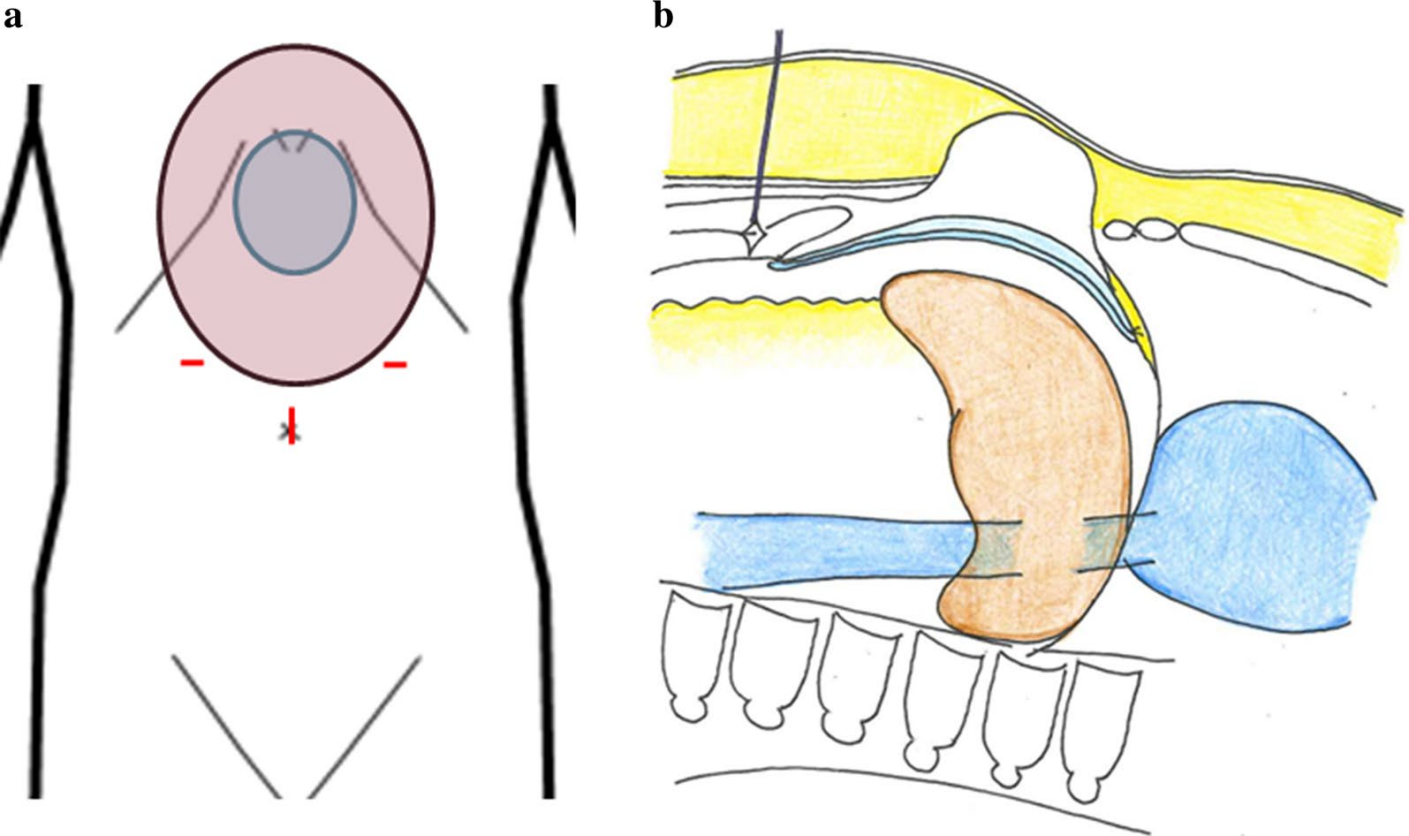
Schema of the surgical procedure. **a** The first trocar was inserted at the umbilicus, and 5-mm trocars were inserted into the upper abdomen bilaterally. The size of the ventral incisional hernia is 6 × 7 cm (light blue). The purple ellipse is the Ventrio™ ST Hernia Patch. **b** Suturing to the falciform ligament and diaphragm, and fixing to the abdominal wall using Lapa-Her-Closure™

**Fig. 3:**
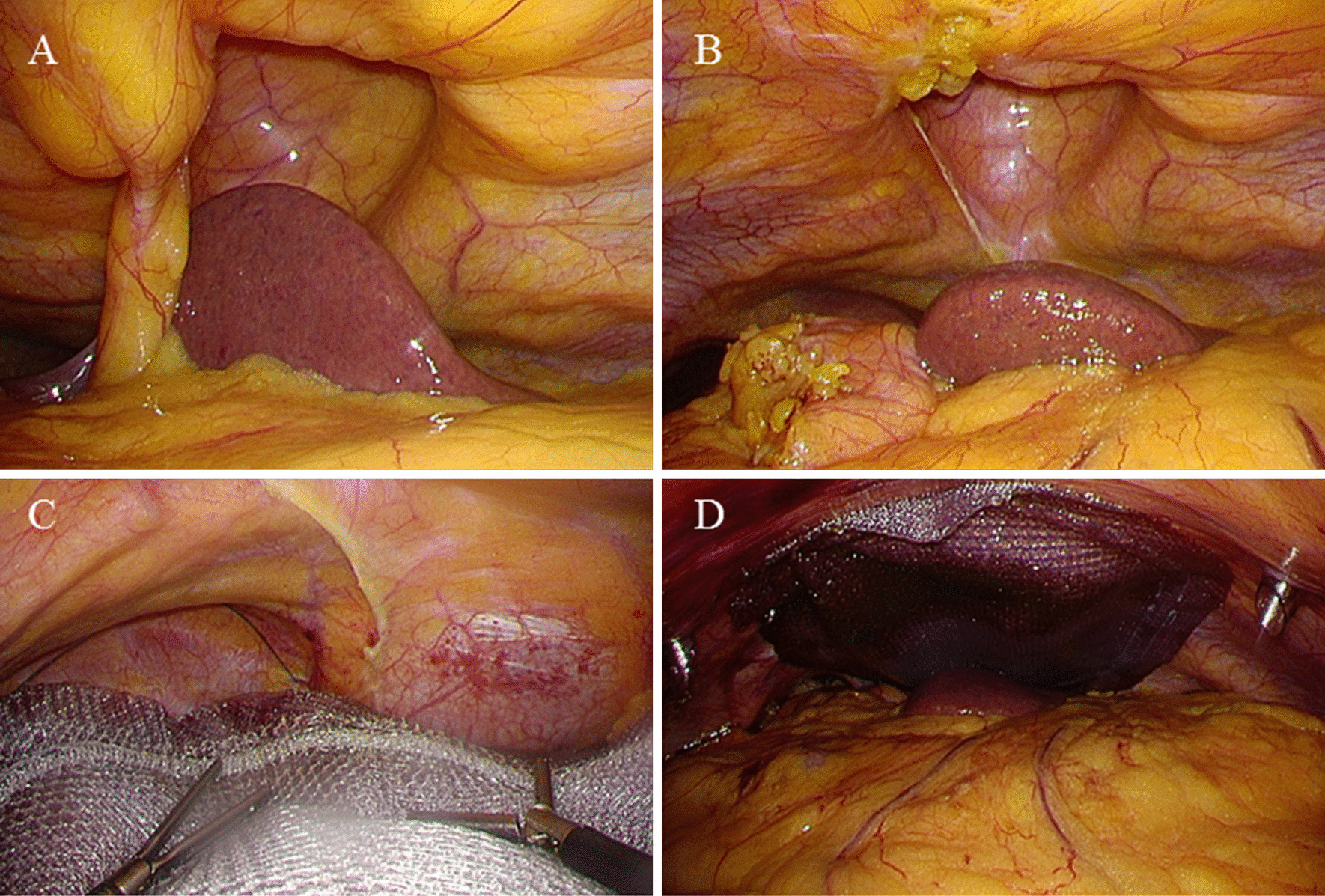
Laparoscopic view of hernia repair. **a** Intraperitoneal view of the hepatic herniation. **b** Dissection of the falciform ligament. **c** Suturing the mesh to the falciform ligament in the good working space. **d** After repair of the ventral incisional hernia

**Fig. 4:**
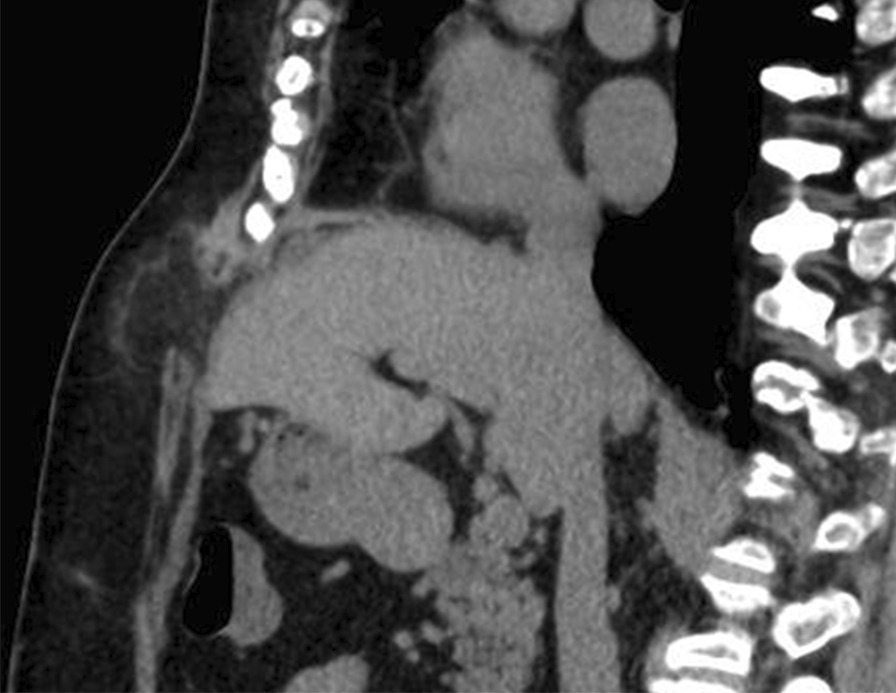
Abdominal computed tomography after 6 months. No recurrence has been observed

A 68-year-old Japanese women presented to our hospital with vomiting and epigastric pain. Her medical history included left ventricular myectomy with median sternotomy for hypertrophic cardiomyopathy 1 year earlier. No abnormal vital signs or cardiovascular function were found. Although she suffered from obesity (body mass index, 37.3 kg/m^2^) and fatty liver, laboratory test results demonstrated normal hemoglobin and hematocrit levels, normal liver enzymes, and normal renal function. Physical examination revealed a 4-cm painless mass protruding through the lower edge of a mid-sternal incision, which gradually expanded 3 months after the cardiac surgery. Abdominal computed tomography revealed left hepatic lobe herniation through a subxiphoid incision (Fig. 1a, b), and showed no other abnormal findings such as cholecystitis. The symptoms were relieved by manual hernia reduction. However, these same symptoms appeared 2 days later, and the patient subsequently required hospitalization. The patient was unable to endure the inconvenient and unpleasant symptoms due to repeated incarcerated hepatic hernias. We considered that surgical intervention was necessary even though she was a high-risk surgical patient (American Society of Anesthesiologists Physical Status Classification, 3) because the symptoms were relieved after hernia reduction. She underwent elective laparoscopic hernia repair. The surgical procedure was performed by modifying the standard techniques followed for other ventral incisional hernias (Fig. 2a, b). The first trocar was inserted at the umbilicus, and 5-mm trocars were inserted into the upper abdomen bilaterally [arranged such that the angle with the deepest sutured part (the falciform ligament) was about 60° without hindering the mesh fixing position on the caudal side]. The falciform ligament of the liver was dissected, a mesh (Ventrio™ ST Hernia Patch, C. R. Bard; Warwick, RI) was spread to completely overlap the incisional hernia (at least 5 cm), and this mesh was sutured to the falciform ligament and diaphragm (five places). In particular, needles (3-0 Polysorb™ GL-222, Covidien, Mansfield, MA, USA) were inserted into the diaphragm shallowly and widely, alternating left and right from the deeper part. The caudal and lateral aspects of the mesh were fixated in a traditional fashion with transfascial sutures placed through the muscular and fascial layers of the abdomen. In addition, the surgery was completed by suturing the mesh to the abdominal wall using a Lapa-Her-Closure™ needle (Hakko Medical; Tokyo, Japan) (Fig. 3a–d). There were no complications, and she was discharged home on postoperative day 5. During the 18-month follow-up, no signs or symptoms of recurrence were observed (Fig. 4).

## Discussion and conclusions

The case reported herein has two important clinical implications: hepatic herniation can occur within a ventral incisional hernia, and laparoscopic repair was useful in the treatment of this type of hernia. Our case is the first report of laparoscopic repair of a ventral incisional hernia with hepatic herniation.

First, prolapse of parenchymal organs such as the liver is rarely found in a ventral incisional hernia. In most cases, the contents of a ventral hernia include the small intestine, colon, and fat or fibrous tissue [3]. According to a recent report, a search using PubMed from January 2000 to January 2019 showed only 20 cases of parenchymal hernias, only nine of which were hepatic herniation [2]. Among them, ventral hernia following median sternotomy or subxiphoid incisional hernia accounted for five out of ten cases of liver prolapse, including our case [4–7]. Hepatic herniation usually protrudes through a large area of the abdominal wall defect and is less likely to be incarcerated. Although it causes nausea and epigastric pain, it rarely leads to serious clinical manifestations. However, of note, there have been reports of a patient with incarceration of the left hepatic lobe causing acute liver failure and hepatic encephalopathy, and a patient with incarceration of the right hepatic lobe causing Budd-Chiari syndrome [8, 9].

Second, this case demonstrated that laparoscopic repair was useful for a ventral incisional hernia with hepatic herniation. A review of the literature revealed no standard treatment strategy for hepatic hernia. Generally speaking, surgical treatment is selected when conservative treatment becomes ineffective. Out of the ten cases of hepatic herniation mentioned previously, four were treated with non-laparoscopic surgery. In open surgery, positioning of the mesh may be carried out through a variety of methods such as onlay, sublay, or underlay. However, laparoscopic repair is based on an underlay approach, which serves to visually reinforce a substantial portion of the abdominal wall compared with open procedures [10]. Therefore, laparoscopic hernia repair is widely used in ventral incisional hernia. However, a hernia located in a challenging site, such as a subxiphoid hernia, requires careful selection of surgical approaches and mesh fixation methods due to high recurrence rates [11–14]. One of the causes of recurrence is the difficulty in suturing and mesh fixation due to complex anatomical structures (the sternum and ribs superior, diaphragm posterior, and high intra-abdominal pressure with sheering forces of the musculature in the upper abdomen). Mesh fixation proves especially difficult, as fixation with tacks to the diaphragm may cause cardiac tamponade and serious complications, and is consequently not recommended [3, 15]. Therefore, there is a risk of recurrence due to mesh displacement. To prevent recurrence, there have been reports indicating that the liver holds the mesh in place after desufflation by the excessive overlap of mesh beyond the uppermost aspect of the defect and that mesh fixation with fibrin glue was useful [3, 16]. However, in our hepatic herniation case, the risk of recurrence was expected to be higher due to extrusion of a parenchymal organ, and we determined that rigid fixation of mesh was required. In this case, safe suturing was possible because adequate working space was secured by the pneumoperitoneum even in a narrow space, under the diaphragm, in addition to the magnifying effect. Furthermore, it was possible to visually confirm absence of mesh sliding during desufflation and the positional relationship between the liver and the mesh, resulting in certainty of repair. We consider that this technique could be achieved with other types of meshes and suturing needles. On the other hand, since there is a risk of complications such as cardiac tamponade or pneumothorax even with this suturing technique to the diaphragmatic area, it is important to avoid cardiovascular and intercostal spaces, to insert needles shallowly and widely, and master safe suturing techniques. Laparoscopic repair for hernia with hepatic herniation might require appropriate patient selection. However, it provides benefits such as decreased postoperative pain, decreased incidence of wound infection, and early rehabilitation [10].

Out of the five cases of subxiphoid hernia with hepatic herniation mentioned previously, four were obese women with fatty liver [4, 6, 7]. Liver prolapse was expected due to the enlarged volume associated with fatty liver and increased intra-abdominal pressure associated with obesity. Although the true incidence is unknown because this type of hernia is generally asymptomatic, the incidence of subxiphoid incisional hernia is reportedly between 1 and 4.2% [12]. Obesity and fatty liver are risk factors in patients with coronary artery disease requiring coronary artery bypass graft surgery [17]. In addition, hernias in these patients are likely to include hepatic herniation. Although conservative treatment is possible in many cases, incarcerated liver may cause liver dysfunction and liver failure. Laparoscopic management of ventral hernias is recommended in cases with obesity [10], and might also be a useful option as an elective surgery for these high-risk surgical patients with hepatic herniation.

In conclusion, hepatic herniation can occur within a ventral incisional hernia, and be repaired laparoscopically by suturing the mesh to the falciform ligament and diaphragm. Although special consideration must be taken into account in patients with previous heart disease and/or hernias following sternotomy, this surgical method is considered suitable due to its minimally invasive nature and increased visibility of the surgical field.

## Data Availability

Not applicable.
